# Relationships among Square Dance, Group Cohesion, Perceived Social Support, and Psychological Capital in 2721 Middle-Aged and Older Adults in China

**DOI:** 10.3390/healthcare11142025

**Published:** 2023-07-14

**Authors:** Yujia Qu, Zhiyuan Liu, Yan Wang, Lei Chang, Hongying Fan

**Affiliations:** 1 School of Art, Beijing Sport University, Beijing 100084, China; 2022210273@bsu.edu.cn; 2 School of Psychology, Beijing Sport University, Beijing 100084, China; 2020011409@bsu.edu.cn; 3 Laboratory of Sports Stress and Adaptation of General Administration of Sport, Beijing 100084, China; 4 Sports Department, China Women’s University, Beijing 100101, China; 0131026@cwu.edu.cn

**Keywords:** square dance, middle-aged and older adults, group cohesion, psychological capital, perceived social support

## Abstract

(1) Background: Aging is a global phenomenon, and China’s aging is extensive and rapid and already at the middle to upper level worldwide. Promoting social interaction and increasing positive psychological qualities in individuals are key components in helping people adapt to the physical and mental changes of the aging process. Among them, how middle-aged and older adults improve their physical and mental health through physical activity is of great concern. (2) Methods: This study measured the physical activity of 2721 middle-aged and elderly square dance participants across China, and structural equation modeling was applied to explore the relationship between square dance exercise and group cohesion as well as the role of perceived social support and psychological capital. (3) Results: The results showed that (a) square dance exercise positively predicts group cohesion among middle-aged and older adults. (b) Perceived social support and psychological capital mediate the relationship between square dance exercise and group cohesion, and the mediating role consists of three pathways: perceived social support alone, psychological capital alone, and perceived social support-psychological capital chain mediation. (c) The mediating effect of psychological capital alone is greater than the mediating effect of perceived social support alone and the mediating effect of the perceived social support-psychological capital chain. (4) Conclusions: This study provides support for the theory and practice of square dance exercise and intervention guidance for increasing positive psychological qualities and group dynamic levels in middle-aged and older adults.

## 1. Introduction

The social problems of aging, such as those related to health and care and loneliness, are becoming increasingly prominent [1]. Physically inactive older adults are more likely to suffer from deteriorating physical and mental health, leading to higher morbidity and mortality [2], as well as experience more serious psychological problems, such as negative emotions, loneliness, weakened social relationships, etc. [3,4]. Physical activity (PA) can be an effective response to aging and is a tool to promote body–mind health in older adults [5], which can provide significant physical, psychological, and social benefits [6,7]. A recent scoping review suggested that PA contributes significantly to the body–mind health of older adults [8].

Square dance is a PA organized by the masses, carried out in public spaces, with popular music as the background and dance movements as the carrier, and with the main purpose of strengthening the body [9]. Square dance is popular with older people for its simple and interesting movements and distinctive rhythm and is the first choice for middle-aged and older adults in China who want to participate in physical activity [10]. Regular participation in square dance exercises can improve several physiological indicators in middle-aged and older adults, as well as effectively increase their self-efficacy, social support, and group cohesion, and improve their positive psychological qualities [11,12].

In recent years, social interaction theory has drawn increasing attention, which focuses on social interaction, manifested as a joint action between individuals, between individuals and groups, and between groups to achieve a common goal [13]. From a sociological perspective, when physiological conditions allow, middle-aged and older adults should actively participate in social interaction activities to improve the negative emotions, such as loneliness, experienced by individuals through new role participation interactions, minimize the distance between themselves and society, effectively improve social connections, and enhance interpersonal relationships [14]. Therefore, middle-aged and older adults participate in sports not only for physical activity but also to improve their social interaction skills. The social interaction in square dance, on the other hand, is oriented to the internal (individual psychological qualities) and external (social interaction relations) mechanisms as the core logic, with the individual-dancer team as the main object [15]. Through participation in square dance activities, it is possible to expand the social contact of middle-aged and older adults and to increase the extensive exchange of information between individuals of different occupations, social statuses, and age stages in terms of work and life, as well as culture and leisure [16]. Given this deduction, this study aims to explore the relationship between square dance exercise and group cohesion among middle-aged and older adults and its internal and external mechanisms of action and to further clarify the individual and group psychological dynamics driving the development of square dance.

### 1.1. Square Dance and Group Cohesion

Group cohesion is a dynamic process that reflects the tendency of a group to stay together and maintain the whole in the pursuit of group goals [17]. Compared to general groups, the climate of sports groups in collective sports can effectively influence the psychological states of its members [18]. Compared to individual sports, group exercise is more conducive to the development of group cohesion by satisfying the basic psychological needs of a sense of competence and relationships with others and by positively experiencing social interactions [19,20].

Social interaction theory suggests that good mutual help relationships between members during participation in exercise affect exercise performance and thus increase the level of group cohesion [21]. Cohesive groups and their individuals demonstrate more positive social interaction behaviors, as highly cohesive groups develop a high level of social identity and promote members’ mutual helping behaviors [22]. In addition, collective psychological ownership facilitates the generation of social interactions within the group [23], as demonstrated by bringing people with the same goals together through the square dance and enabling identity recognition from it, reaching a shift from “I” to “we”. This shift increases the frequency of intimacy between social interactions and contributes to group cohesion [24].

Strong group cohesion increases the attraction of and satisfaction between the sports group and its members, generates a strong sense of mission and collaboration, and further increases the motivation and creativity of members [25]. Square dance is considered a special interactive ritual from which the sense of belonging and group cohesion among its members can be reflected. Studies have confirmed that collectivism plays a role in the formation and development of Chinese square dance organizations. In summary, square dance exercises can promote group cohesion to a certain extent, while cohesion is also inseparable from square dance exercise.

### 1.2. The Relationship between Perceived Social Support and Group Cohesion

Social support is a key determinant of positive aging [26]. Research has shown that perceived social support is more meaningful in predicting individuals’ psychological well-being than other types of social support [27], so the present study explores the concept of perceived social support, which is an individual’s subjective feelings and emotional experiences of the social support he or she may receive during interpersonal interactions in social life. Emotional interaction is an important type of interaction in social interaction theory, where intimate groups such as family and friends are used to meet the emotional needs of individuals. Through square dance exercises, older adults can gain support and understanding from their families and dance friends, which is more in line with the social and recreational value of square dance.

According to the main effect and buffering model of social support, social support has a generally beneficial effect on an individual’s mental health development and can buffer the negative effects of stressful events on an individuals’ physical and mental condition and maintain good emotional states [28]. Previous empirical studies of middle-aged and older adults have shown that participation in group exercise, such as square dance, helps increase individuals’ positive emotions, expands their interpersonal channels in daily life, satisfies their need for social interaction and emotional communication, and increases their likelihood of receiving social support [29]. In addition, perceived social support positively contributes to increased group cohesion and increased adherence to PA participation [30,31,32]. High levels of perceived social support are highly associated with good physical and mental health and task cohesion [33]. In summary, the more social support people perceive through square dance, the stronger the team’s task accomplishment and attraction among members, which can further increase group cohesion.

### 1.3. The Relationship between Psychological Capital and Group Cohesion

Psychological capital (Psycap) comprises the positive psychological elements that can be developed and improved by an individual, is reflected in the long-term, cumulative positive psychological state that an individual exhibits during the process of growth and development, and includes four main elements: self-efficacy (an individual’s measurement and assessment of whether he or she can do something successfully), optimism (a positive prediction of the current situation and future), hope (an individual’s belief that he or she can work hard toward a goal and achieve success), and psychological resilience (an individual’s confidence in doing something and the spirit of rising to the challenge) [34]. These four elements are the most typical representatives of positive psychological resources emphasized by positive organizational behavior (POB), which can effectively leverage the strengths of group members positively and improve overall organizational performance [35] and are closely related to group dynamics and cohesion. At the same time, reciprocity is a fundamental principle followed by social interaction theory, and individuals’ perceived organizational support motivates them to participate in group sports and improves positive psychological qualities while helping strengthen interpersonal skills, thus, building social relationships and contributing to further group cohesion [36].

Psycap is a psychological resource that can be effectively increased through group activities [37]. In this study, this was demonstrated by the positive social interactions stimulated by the square dance exercise; the development of individual hope, optimism, and psychological resilience; the increase in self-efficacy, and thus the improvement in psycap [34,38]. At the same time, the effective development of psycap can enhance emotional communication and interpersonal communication skills, which further influence team value congruence and group cohesion [39]. In conclusion, psycap is a concept that reflects the dynamics of changes in the living conditions of middle-aged and older adults and is a relatively stable but plastic psychological quality. Square dance exercise could effectively promote the development of individual psychological qualities and develop psycap to further improve group cohesion.

### 1.4. The Serial Multiple Mediation Mechanism of Perceived Social Support and Psychological Capital

Resource conservation theory suggests that social support is a potential psychological resource [40]. Social support, especially social interactive social support, is a protective factor for the psychological health of middle-aged and older adults and is effective in strengthening social interactions, improving individual positive emotions, and creating a good atmosphere for exercise utilizing group physical activities [41]. Psycap is a psychological resource that can be increased in various ways (e.g., social relationships) and can make individuals feel positive psychological qualities and psychological energy, such as hope and self-confidence [35,42]. It can also stimulate positive emotions and encourage individuals to actively participate in social activities [43]. Previous research has found a link between perceived social support and psycap, with individuals perceiving higher levels of positive psycap when they perceive more social support [44].

Empirical studies on middle-aged and older adults have shown a significant positive correlation between perceived social support and psycap. A five-month online continuous coaching health intervention and three months of moderate-to-vigorous PA can positively influence psycap and perceived social support factors [45]. In addition, perceived social support can effectively increase factors such as self-efficacy, hope, optimism, psychological resilience, and subjective well-being, which can effectively increase the level of positive psycap [46,47].

As previously mentioned, PA is an important way to increase an individual’s perceived social support, and perceived social support is also a key factor influencing an individual’s positive mood. The group form of square dance helps older people improve their social participation and psychological resilience, establish hope and optimism, increase their sense of self-efficacy and value, and finally, improve the level of group cohesion.

### 1.5. The Present Study

Most previous studies have focused on the effect of square dance exercise on improving individuals’ mental health [48,49]. However, square dance is more oriented to daily exercise in groups, which can provide middle-aged and older adults with a relaxed and enjoyable exercise atmosphere and opportunities to expand their social interactions during group exercise [50]. Therefore, the social group nature of square dance exercise also deserves attention. However, the mechanisms by which the group effect of square dance exercise can be promoted are still insufficiently understood, and studies based on psycap and perceived social support as mediating variables are relatively limited. At the same time, few cross-sectional surveys have been conducted on large samples of square dance. Because of this, the present study aims to investigate the relationship between square dance exercise and the cohesion of middle-aged and older adults and the underlying mechanisms of psycap and perceived social support to enrich social interaction theory and provide a theoretical basis for future practice and intervention studies. Therefore, the hypotheses of this study are as follows.

**H1.**  *Square dance exercise positively predicts group cohesion in middle-aged and older adults.*

**H2.**  *Perceived social support mediates the relationship between square dance exercise and group cohesion.*

**H3.**  *Psychological capital mediates the relationship between square dance exercise and group cohesion.*

**H4.**  *Perceived social support and psychological capital have a serial mediating effect on the association between square dance exercise and group cohesion.*

## 2. Materials and Methods

### 2.1. Participants

The data were collected from 10 April to 10 May 2023 through a web-based survey hosted by Wenjuanxing (https://www.wjx.cn/). We monitored the IP addresses of the respondents to avoid multiple responses. The questionnaire was distributed across 32 provinces, autonomous regions, and municipalities, including Beijing, Jiangsu, Guangdong, Fujian, Inner Mongolia, Shanghai, Qinghai, Shandong, Yunnan, Hunan, Zhejiang, Chongqing, Sichuan, Hebei, Anhui, and Taiwan, with a wide geographical distribution of the sample. The initial dataset consisted of 2721 respondents. The validation of data was taken into consideration; some samples were deleted due to the limitation of age, time, accuracy of Body Mass Index (BMI), and questionnaire completion status during the analysis. Participants were required to be middle-aged or 45 years or older, and through screening, we excluded 56 of them because they were younger than 45 years old. In addition, 3 were excluded because their BMI (height and weight) were filled in incorrectly, 151 because their response times were too short, and 3 because they did not complete the entire survey. In conclusion, a total of 2428 respondents were included in this study, with a questionnaire validity rate of 89.23%. The mean age of the participants was 60.62 ± 6.68 years, with 117 males (4.8%) and 2311 females (95.2%).

### 2.2. Measures

- Demographic information—Demographic information obtained in the questionnaire included participants’ sex, age, monthly income, BMI, intensity and duration of square dance exercise, and group size.
- Square dance exercise—Square dance exercise was assessed using the Physical Activity Rating Scale-3 (PARS-3) to examine individuals’ actual participation in square dance exercise in the last month [51]. In this study, Liang’s translation of the original PARS-3 was used [52]. The questionnaire contains three questions, one each on exercise intensity, duration, and frequency of activity. The calculation formula is: Amount of exercise = intensity × time × frequency, the scoring method is: intensity and frequency are scored from 1 to 5 points; time is scored from 0 to 4 points. The highest score is 100, and the lowest is 0. Regarding the evaluation criteria for the amount of exercise: <19 is categorized as low volume; 20–42 is categorized as medium volume; >43 is categorized as large volume. Cronbach’s α was 0.65, and the retest reliability of each item on the scale was 0.83.
- Perceived social support—Perceived social support was assessed using the Perceived Social Support Scale (PSSS), which is used to examine individuals’ perceived social support from others [53]. In this study, the original PSSS was adjusted to Chinese by Jiang [54]. The questionnaire contains three dimensions with four items each: family support, friend support, and other support. The scale is scored on a 7-point scale (1 = strongly disagree, 7 = strongly agree). In this study, Cronbach’s α for this scale was 0.95.
- Psychological capital—Psycap was assessed using the Positive Psychological Questionnaire (PPQ), which is used to examine individual psycap, i.e., the positive psychological states that people exhibit when they accomplish tasks and achievements [55]. In this study, the original PPQ was adjusted to Chinese by Zhang [56]. The questionnaire contains four dimensions with six dimensions each: self-efficacy, hope, optimism, and mental toughness. It is scored on a 7-point scale (1 = strongly disagree, 7 = strongly agree). Items are averaged, with higher mean scores indicating a higher psycap. In this study, Cronbach’s α of the scale was 0.91.
- Group cohesion—Group cohesion was assessed using the Group Environment Questionnaire (GEQ), which is used to reflect the overall perception of the group and the degree of personal attraction of the members of a square dance organization in the pursuit of exercise goals [57]. In this study, the Chinese version of the GEQ translated and revised by Ma [58] was used to conduct the survey. The questionnaire contains four dimensions: interaction congruence, interaction attractiveness, task congruence, and task attractiveness. The scale has a total of 15 items and is scored on a 7-point scale (1 = strongly disagree, 7 = strongly agree). In this study, Cronbach’s α for the scale was 0.90.

### 2.3. Statistical Analysis

The statistical analysis of the data was carried out with SPSS 25.0 and Mplus 8.3, with α = 0.05 as the statistically significant level. SPSS 25.0 was used to calculate and describe the distribution of each variable and their correlation; Mplus 8.3 was used for confirmatory factor analysis (CFA) and to test the mediating effect and path differences proposed in the research hypotheses.

Meanwhile, the scale items used in this study have more items, so more parameters are required for modeling; thus, the items are packaged. Wu and Wen [59] suggested that the internal-consistency approach is preferred for packaging multidimensional scales, i.e., packaging items of the same dimension into one indicator. Therefore, the PARS-3 and PSSS used in this study each have 3 dimensions and are packaged into 3 indicators, while the PPQ and GEQ each have 4 dimensions and are packaged into 4 indicators. Additionally, it should be noted that the larger the sample, the more sensitive the cardinality value, which greatly increases the probability of incorrectly rejecting the model [60], and it is also necessary to combine other indices to examine the true model fit [61].

Structural equation modeling was used to test the mediating roles of perceived social support and psycap in the relationship between square dance exercise and group cohesion. The predictor variable was square dance exercise, the mediating variables were perceived social support and psycap, and the outcome variable was group cohesion. Significance tests were conducted using the bias-corrected bootstrap method with 5000 replicate samples with putbacks, and the mediating effect was tested for significance based on whether the 95% confidence interval contained 0. If the confidence interval did not contain 0, the mediating effect was significant, and if it contained 0, it was not significant [62].

## 3. Results

### 3.1. Preliminary Results

Table 1 shows the demographic information, means, and SDs for the intensity and duration of square dance, perceived social support, psycap and group cohesion for group sizes less and more than 25 people.

### 3.2. Common Method Variance Test

The data in this study are collected from self-report measures, which can cause common method variance (CMV). The necessary controls were carried out during the test process. To reduce the variance, Harman’s single-factor test was performed for statistical control before data analysis, and all variable items were subjected to unrotated principal component factor analysis [63]. The results indicated that the variance explained by the first factor was 35.93%, less than the critical value of 40% [64]. Additionally, further CFA was used, and the results showed that the single factor model fit was poor: χ^2^/*df* = 31.25, RMSEA = 0.112, CFI = 0.546, TLI = 0.529, testing the CMV hypothesis more precisely. Combining the two tests, there was no significant CMV in the data.

### 3.3. Descriptive Statistics and Related Analysis

The average, standard deviation and Pearson correlation matrix of each variable are listed in Table 2. There were significant positive correlations between square dance exercise and perceived social support, psycap, and group cohesion (*r* = 0.031–0.916, *ps* < 0.01). The correlation analysis of each dimension of the scale revealed that, except for the mental toughness dimension of psycap and the friend support dimension of perceived social support, which were not significantly correlated (*ps* > 0.05), square dance exercise was significantly and positively correlated with perceived social support (*r* = 0.707–0.749, *ps* < 0.01), psycap (*r* = 0.055–0.798. *ps* < 0.01) and cohesion (*r* = 0.403–0.916, *ps* < 0.01). Perceived social support was significantly and positively correlated with psycap (*r* = 0.055–0.798, *ps* < 0.01), and perceived social support, psycap (*r* = 0.707–0.916, *ps* < 0.01) and cohesion (*r* = 0.055–0.916, *ps* < 0.01) were significantly and positively correlated. The above results reveal that it is suitable for further mediating effects analysis.

### 3.4. Differential Analysis

After completing the descriptive statistics and related analysis, we used *t*-tests to further explore differences in each variable among middle-aged and older adults across control variables, such as age, sex, monthly income, and marital status, as shown in Table 3. The results show that: (1) age, there was a significant difference in group cohesion (*ps* < 0.001) between middle-aged groups cohesion levels and older groups; (2) sex, there was a significant difference in square dance exercise group cohesion (*ps* < 0.05) between male’s physical activity level and female’s, between female’s group cohesion level and male’s; (3) monthly income, there was a significant difference between monthly income in terms of group cohesion (*ps* < 0.001) and in terms of perceived social support (*ps* < 0.05), with higher levels of group cohesion and perceived social support among middle-aged and older adults with a monthly income of less than 3500 yuan than among those with a monthly income of more than 3500 yuan; (4) marital status, there were no significant differences among the variables due to marital status (*ps* > 0.05).

### 3.5. The Serial Mediating Effect of Perceived Social Support and Psychological Capital

Before testing for mediating effects, the direct effect of the square dance exercise on group cohesion was first tested, and the model had a good fit index, χ^2^/*df* = 14.33, CFI = 0.979, TLI = 0.967, RMSEA = 0.074, SRMR = 0.028. The results indicate that square dance exercise significantly and positively predicts group cohesion (*β* = 0.204, *p* < 0.001).

Next, models 1 and 2 were developed with perceived social support and psycap as single mediating variables, respectively. The results show a good fit (model 1: χ^2^/*df* = 12.76, CFI = 0.974, TLI = 0.963, RMSEA = 0.070, SRMR = 0.033; model 2: χ^2^/*df* = 20.53, CFI = 0.946, TLI = 0.928, RMSEA = 0.090, SRMR = 0.039); the mediating effect was tested by using the bias-corrected bootstrap method with 5000 repeated samples, and the mediating effect of perceived social support was 0.406, 95% *CI* [0.094, 0.728], with an effect size of 18.3%; and the mediating effect of psycap was 1.209, 95% *CI* [0.860, 1.616], with an effect size of 58.1%.

**Figure 1:**
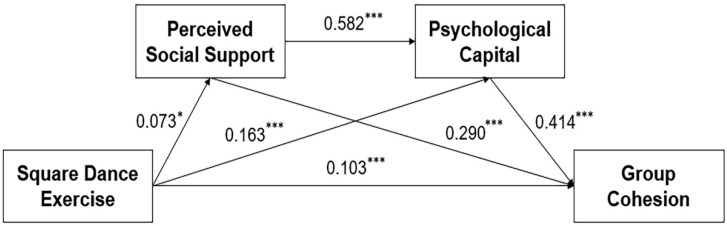
Research hypothesis model (*N* = 2428). Note: * *p* < 0.05, *** *p* < 0.001; The data are standardized path coefficients.

Next, model 3 (serial mediation) was developed. The results showed a good fit with fit indices of χ^2^/*df* = 16.04, CFI = 0.95, TLI = 0.936, RMSEA = 0.079, SRMR = 0.039. In model 3, all path coefficients were significant, and the standardized values of each coefficient are shown in Figure 1.

The path diagrams in Figure 1 show that (1) square dance exercise (*β* = 0.103, *p* < 0.001) can significantly and positively predict group cohesion, indicating that the greater the square dance exercise is, the higher the group cohesion of the middle-aged and older adults; (2) square dance exercise *(β* = 0.073, *p* = 0.013) significantly and positively predicts perceived social support, indicating that the greater the square dance exercise is, the more social support elderly people feel; (3) square dance exercise (*β* = 0.163, *p* < 0.001) significantly and positively predicts psycap, indicating that the greater the square dance exercise is, the more adequate the positive psycap; and (4) both perceived social support (*β* = 0.290, *p* < 0.001) and psycap (*β* = 0.414, *p* < 0.001) significantly and positively predict group cohesion. (5) Perceived social support (*β* = 0.582, *p* < 0.001) significantly and positively predicts psycap. Using the bias-corrected bootstrap method with 5000 repeated samples to test each path of the serial mediation model, the mediating effect of perceived social support was 0.209, 95% *CI* [0.054, 0.402], with an effect size of 10.1%; the mediating effect of psycap was 0.669, 95% *CI* [0.456, 0.944], with an effect size of 32.3%; and the serial mediated effect was 0.174, 95% *CI* [0.038, 0.338], with an effect size of 8.4%. The above results show that perceived social support and psycap play a serial mediating role in the relationship between square dance exercise and group cohesion. The details of each pathway situation of the model are shown in Table 4.

Finally, we further compared the differences between the three mediating paths and found that the mediating effect of path two (square dance exercise-psychological capital-group cohesion) was significantly larger than that of path one (square dance exercise-perceived social support-group cohesion), with 95% *CI* [0.159, 0.770], *t* = 2.970, *p* = 0.003. Additionally, the mediating effect of path two (square dance exercise-psychological capital-group cohesion) was significantly larger than that of path three (square dance exercise-perceived social support). The mediating effect of path two (square dance exercise-psychological capital-group cohesion) was significantly larger than that of path three (square dance exercise-perceived social support-group cohesion), 95% *CI* [0.239, 0.779], *t* = 2.970, *p* < 0.001. Thus, psycap plays a more critical role in the relationship between square dance exercise and group cohesion.

## 4. Discussion

This study systematically investigates the relationship between square dance exercise and group cohesion and its inner psychological mechanisms among middle-aged and older adults, and it is the largest cross-sectional survey of square dance projects in China to date. Perceived social support is a concrete manifestation of external mechanisms in social interaction theory and can increase social interaction ability by providing emotional and behavioral support [65]. Meanwhile, psycap can be transformed from external mechanisms to internal resources [66] and is closely related to group indicators such as group behavior style and identity. Again, because of the group nature of square dance, we also included quantities such as exercise intensity, group size, and group exercise duration in the demographic information to more fully depict the current status of group exercise in square dance among Chinese middle-aged and older adults. This study supports the positive association between square dance exercise and cohesion. At the same time, a serial mediation model was eventually developed and verified H1, H2, H3, and H4.

In addition, in terms of the composition of the participants, the study supported the fact that the majority of square dance participants were female, which is consistent with previous findings [67]. Because research showed that improvements in mood and social function (i.e., feeling less depressed and more confident; becoming friends with more dancers) were greater for females than for males [68] and more in line with female physical and psychological needs [69]. At the same time, we did not exclude the 117 male participants in this study who participated and reported on the survey, which provided a theoretical basis for future studies on male square dancers.

### 4.1. The Direct Relationship between Square Dance and Group Cohesion

Cohesion is an important factor in people’s adherence to regular PA, and group exercise. Additionally, the present study found that the higher the level of group cohesion for moderately intense square dance exercise, the more years of exercise and the larger the group size, which is consistent with previous findings [70]. This indicates that exercise intensity, exercise duration, and group size are the core elements that promote the development of square dance, which can effectively improve the coordinated development of all team factors and further promote cohesion.

### 4.2. The Intermediary Role of Perceived Social Support

Social interaction theory states that social relationships can improve mental health through physical activity [71]. Therefore, PA can be effective in developing social relationships and thus improve individual psychological health. Previous findings are consistent with the results of the present study that PA increases the level of perceived social support in elderly groups [72] and suggests that social connectedness and interpersonal contact are the main reasons for participation in group sports [73,74].

At the same time, exercise can foster good social relationships and friendship networks, thus increasing group cohesion [75]. As a group sport with a good atmosphere, square dance can promote high-frequency social communication among members and expand group attractiveness, thus, helping improve the level of perceived social support and further increase group cohesion. However, this study found that the mediation effect of perceived social support alone was the lowest among the paths. A possible reason for this is that the friend support dimension was not significantly correlated with square dance exercise. First, we suggest that family support plays a dominant role in perceived social support [76]. Older adults who had participated in PA for more years scored higher on the family support dimension [77]. Second, this investigation was conducted during the COVID-19 outbreak, when the social environment may have hindered social interactions, and the inevitable psycho-emotional impact of the virus on individuals may also have led to a lower tendency to socialize [78]. Despite these results, we replicated the findings of most previous studies exploring the relationship between the two that perceived that social support has a positive effect on PA and health behaviors [79,80]. Of course, the pathway of square dance exercise to improve perceived social support in middle-aged and older adults needs to be further validated by subsequent studies.

### 4.3. The Intermediary Role of Psychological Capital

Social communication is considered an important factor influencing positive emotions and an important way for middle-aged and older adults to seek psychological communication. Through social interaction, individuals can increase their self-efficacy, maintain positive emotional states, and promote group behavior [81]. Previous studies have confirmed that PA can improve self-efficacy, sense of belonging, and achievement, i.e., psycap can be increased, which is consistent with the results of the present study. Square dance is a moderate-intensity aerobic exercise [82], and moderate-intensity aerobic exercise provides the best psychological benefits [83]. We further verified that moderate-intensity square dance exercise improves individuals’ positive emotional experiences and can effectively increase their psycap. The increase in positive psycap is crucial for improving the group cohesion of middle-aged and older square dance participants.

The mediating effect of psycap alone was the largest among the paths, possibly because with age and experience, older people who participated in square dance exercise for a long time would have stronger psychological adaptability and be more likely to acquire optimism and hopefulness, leading to strong relationships among exercise, psycap, and cohesion. During square dance exercise, members teach and learn from each other through physical and verbal communication, which facilitates motivation, increases self-efficacy, improves optimistic attributions and hopefulness, satisfies social and emotional needs among members, and further promotes psycap [84]. In addition, square dance exercise was significantly and negatively correlated with the psychological resilience dimension of psycap, which differs from previous findings on improving psychological resilience through exercise [85,86]. This may be because square dance includes the characteristics of group, freedom, openness, and interaction, and the purpose of the exercise is mainly to entertain the body and mind and maintain good physical condition with relatively low exercise intensity and load levels. Compared to competitive sports, which aim to win, square dance requires less effort to overcome athletic difficulties and psychological stress. In addition, research has shown that psycap is a core construct whose combined effect is significantly higher than the sum of the four subdimensions [87]. Therefore, we believe that the overall concept of psycap should be used to provide an integrated account and explore the mechanisms of action.

### 4.4. The Serial Multiple Mediation Mechanism of Perceived Social Support and Psychological Capital

Many previous studies have confirmed that perceived social support can have a positive impact on psycap [88,89], while social support has been shown to increase self-efficacy and hope in middle-aged and older groups, thus increasing their psycap [90]. Based on the reciprocity principle of social interaction theory, increasing social interactions can foster optimism, renew hope in life, and increase the level of psycap, thus, contributing to the health status of older adults. Individuals with high self-efficacy have more self-confidence and the ability to actively deal with interpersonal relationships in social life and strive to achieve their social values [91]. The direct and indirect effects of perceived social support on psycap can also be confirmed in practice.

Square dance exercise not only provides external social resources (e.g., peer mutual help and family support) and increases social interaction ability but also helps improve internal individual psychological resources (e.g., self-efficacy, hope, and optimism) and meet the self-actualization needs of middle-aged and elderly groups, and the two can transform each other and develop in a coordinated manner, thus further increasing the level of group cohesion. In addition, although the effect of serial mediation in this study was not as large as the separate path effect of psycap, the effect was still statistically significant, indicating that the joint role played by perceived social support and psycap cannot be ignored. We also look forward to future empirical and practical evidence.

### 4.5. Limitations and Future Research Directions

There are also some limitations in this study. First, this study is a cross-sectional survey and lacks long-term follow-up of the participants. Although the previous theoretical and empirical results provide a certain basis for this study, it is difficult to infer the causal association between the variables, and future studies need to combine experimental and follow-up studies for further multidimensional analysis and validation to reveal the mechanism of the variables in more depth. Second, considering the specificity and complexity of middle-aged and older adults, the questionnaire used was not targeted at the elderly population, which may have caused measurement validity problems and may have affected the predictive effect of the model. Finally, in addition to the indicators included in this study, in reality, other socio-demographic variables such as education level, having children and/or grandchildren, race and ethnicity, medical history of chronic disease, and other important psychological variables such as anxiety, depression, loneliness, motor motivation, temperament type, and personality traits cannot be excluded. It could be included in future related research. Future variable selection and model construction, especially the influence of multidimensional complex concepts and potential factors on the relationship between variables, remain to be tested experimentally and practically, and these need to be considered.

### 4.6. Implications

Theoretical implications: This study revealed the important roles of square dance exercise, perceived social support, and psycap on the cohesion of middle-aged and older adults and explored the serial mediation model of perceived social support and psycap, further elucidating the internal and external mechanisms of both. In terms of internal psychological qualities, efforts should be made to improve self-efficacy, optimism, and other aspects of psycap to further increase the improvement in individual positive psychological emotions [92]. In terms of the external context, it is important to care about the social support of middle-aged and older adults, especially for those in special situations, such as living in an empty nest, being widowed, and living alone [93,94]. In summary, this study explored the effects of both internal individual psychological qualities and external social relationships on group cohesion, further extending the findings of social interaction theory in the field of group social psychology research and providing a rich theoretical reference value for empirical studies of square dance exercise interventions for middle-aged and older adults.

Practical implications: This study has important practical implications for intervention research on increasing positive psychological qualities and group dynamics levels in middle-aged and older adults. Research has shown that after adulthood, there is a multifaceted observable decline in physical condition and physiological functioning [95] and that specific disorders and specific mental health problems in middle-aged and older adults can lead to a proliferation of illnesses and accelerated aging [96,97]. However, we suggest that psycap and group cohesion are remedies to improve the physical and mental conditions of older adults, both of which can be shaped by PA and group exercise interventions to improve individual psycap and increase group cohesion [98]. This provides new ideas for interventions to achieve the goal of active aging early while safeguarding healthy aging.

## 5. Conclusions

This study is the first evidence of square dance exercise in China to generate the largest sample size in a cross-sectional study. The findings emphasize that (1) square dance exercise positively predicts group cohesion among middle-aged and older adults; (2) perceived social support and psycap mediate the relationship between square dance exercise and group cohesion, and the mediating effect consists of three pathways—perceived social support alone, psycap alone, and the serial mediation of perceived social support and psycap; (3) the mediating effect of psycap alone is larger than the mediating effect of perceived social support alone and serial mediation. Based on social interaction theory, this study aims to emphasize that square dance exercise can increase cohesion in elderly groups while improving individual positive psychological qualities and increasing social participation among group members.

## Figures and Tables

**Table 1:** Demographic information of participants.

	Total *N* = 2428	Group Size < 25 *N* = 1279 (52.7%)	Group Size > 25 *N* = 1149 (47.3%)	*t*
Age (in years)	60.62 ± 6.68	60.91 ± 6.79	60.31 ± 6.53	2.190
Sex				
female	2311 (95.2%)	1231 (96.2%)	1080 (94.0%)	
male	117 (4.8%)	48 (3.8%)	69 (6.0%)	
BMI	24.00 ± 48.75	22.97 ± 2.80	25.14 ± 70.81	
Monthly income				
<3500 yuan	1212 (49.9%)	662 (51.8%)	550 (47.9%)	
>3500 yuan	1216 (50.1%)	617 (48.2%)	599 (32.1%)	
Square dance exercise intensity	21.5 ± 14.43	19.47 ± 13.59	23.86 ± 14.97	
Square dance exercise duration				
<5 years	829 (34.1%)	519 (40.6%)	760 (59.4%)	
>5 years	1599 (65.9%)	310 (27.0%)	839 (73.0%)	
PSSS	69.32 ± 12.23	68.41 ± 12.65	70.39 ± 11.55	−4.020 ***
PSSS-FS_1_	22.73 ± 4.53	22.42 ± 4.63	23.08 ± 4.35	−3.581 ***
PSSS-FS_2_	23.82 ± 4.25	23.55 ± 4.40	24.14 ± 4.03	−3.422 ***
PSSS-OS	22.77 ± 4.50	22.43 ± 4.64	23.17 ± 4.28	−4.071 ***
PPQ	139.80 ± 19.78	137.58 ± 20.68	142.37 ± 18.09	−6.036 ***
PPQ-SE	39.24 ± 7.57	38.31 ± 7.92	40.31 ± 6.96	−6.575 ***
PPQ-Resilience	30.43 ± 6.34	30.23 ± 6.33	30.68 ± 6.28	−1.782
PPQ-Hope	34.27 ± 5.83	33.65 ± 6.00	34.98 ± 5.48	−5.677 ***
PPQ-Optimism	35.85 ± 5.48	35.40 ± 5.72	36.39 ± 5.05	−4.522 ***
GEQ	88.78 ± 11.81	87.05 ± 12.72	90.71 ± 10.39	−7.719 ***
ATG-S	25.15 ± 3.54	24.69 ± 3.95	25.66 ± 2.93	−6.841 ***
ATG-T	18.90 ± 2.67	18.56 ± 2.96	19.28 ± 2.25	−6.695 ***
GI-S	20.23 ± 4.10	19.82 ± 4.01	20.69 ± 4.14	−5.278 ***
GI-T	24.50 ± 3.66	23.99 ± 3.96	25.08 ± 3.20	−7.430 ***

Note: BMI, Body Mass Index; FS_1_, Friend support; FS_2_, Family support; OS, Other support; SE, Self-efficacy; ATG-S, Individual attraction to group-social; ATG-T; Individual attraction to group-task; GI-S, Group integration-social; GI-T, Group integration-task; *** *p* < 0.001.

**Table 2:** Descriptive statistics of and associations among the variables (*N* = 2428).

Variables	*M*	*SD*	1	2	3	4	5	6	7	8	9	10	11
1. PARS-3	21.55	14.43	_										
2. PSSS-FS_1_	22.74	4.52	0.031	_									
3. PSSS-FS_2_	23.84	4.24	0.080 **	0.707 **	_								
4. PSSS-OS	22.78	4.49	0.055 **	0.847 **	0.749 **	_							
5. PPQ-SE	39.25	7.55	0.158 **	0.422 **	0.402 **	0.443 **	_						
6. PPQ-Resilience	30.44	6.31	−0.003	0.111 **	0.070 **	0.122 **	0.291 **	_					
7. PPQ-Hope	34.28	5.80	0.144 **	0.424 **	0.456 **	0.465 **	0.759 **	0.055 **	_				
8. PPQ-Optimism	35.87	5.44	0.115 **	0.499 **	0.511 **	0.535 **	0.740 **	0.235 **	0.798 **	_			
9. GEQ-ATG-S	25.148	3.54	0.145 **	0.463 **	0.440 **	0.477 **	0.502 **	0.132 **	0.481 **	0.514 **	_		
10. GEQ-ATG-T	18.90	2.67	0.156 **	0.430 **	0.430 **	0.450 **	0.507 **	0.116 **	0.498 **	0.518 **	0.916 **	_	
11. GEQ-GI-S	20.23	4.10	0.128 **	0.271 **	0.239 **	0.269 **	0.250 **	−0.118 **	0.319 **	0.278 **	0.424 **	0.403 **	_
12. GEQ-GI-T	24.50	3.66	0.109 **	0.515 **	0.486 **	0.534 **	0.475 **	0.105 **	0.498 **	0.517 **	0.813 **	0.803 **	0.485 **

Note: ** *p* < 0.01. Abbreviations: FS_1_, Friend support; FS_2_, Family support; OS, Other support; SE, Self-efficacy; ATG-S, Individual attraction to group-social; ATG-T, Individual attraction to group-task; GI-S, Group integration-social; GI-T, Group integration-task.

**Table 3:** Independent samples *t*-test for each variable (*N* = 2428).

Variables	Classification	Square Dance (*M* ± *SD*)	*t*	PSSS (*M* ± *SD*)	*t*	PPQ (*M* ± *SD*)	*t*	GEQ (*M* ± *SD*)	*t*
Age (in years)	<60	21.93 ± 14.90	−0.55	69.41 ± 12.32	0.30	140.33 ± 19.03	1.28	89.65 ± 11.76	3.86 ***
	>60	21.15 ± 13.87		69.26 ± 12.17		139.31 ± 20.30		87.80 ± 11.79	
Sex	Male	27.53 ± 18.06	4.65 **	70.31 ± 11.76	0.88	140.03 ± 20.93	0.10	86.13 ± 13.68	−2.48 **
	Female	21.24 ± 14.15		69.30 ± 12.20		139.84 ± 19.58		88.91 ± 11.70	
Monthly income (yuan)	<3500	21.33 ± 14.77	−0.73	70.04 ± 12.54	2.81 **	140.48 ± 20.16	1.60	89.96 ± 11.32	4.94 ***
	>3500	21.76 ± 14.07		68.66 ± 11.78		139.21 ± 19.10		87.60 ± 12.17	
Marital status	Unmarried	24.33 ± 16.52	0.67	69.92 ± 11.68	0.16	141.66 ± 11.56	0.32	89.25 ± 11.28	0.14
	Married	21.53 ± 14.41		69.93 ± 12.19		139.84 ± 19.67		88.77 ± 11.81	

Note: ** *p* < 0.01, *** *p* < 0.001.

**Table 4:** Bias-corrected bootstrap results for each path coefficient of the model.

Intermediary Process	Effect Type	Effect Value	Bootstrapped LLCI ^a^	Bootstrapped ULCI ^a^	Effect Size (%)
Square dance—Perceived social support—Group cohesion	Mediating effect	0.406	0.094	0.728	18.3
Square dance—Psycap—Group cohesion	Mediating effect	1.209	0.860	1.616	58.1
Square dance—Perceived social support—Psycap—Group cohesion	Serial mediating effect	0.209	0.054	0.402	8.4
	Mediating effect of PSSS	0.669	0.456	0.944	10.1
	Mediating effect of Psycap	0.174	0.038	0.338	32.3
Total effect		2.074	1.333	2.796	

Note: ^a^ Boot LLCI and Boot ULCI refer to the 95% confidence of the indirect effect estimated by the bias-corrected bootstrap method and the upper and lower limits of the intervals, respectively.

## Data Availability

The authors acknowledge that all data generated or analyzed during this study are included in this published article. The datasets generated during and/or analyzed during the current study are available from the corresponding author(s) on reasonable request.
